# Treatment of atrial fibrillation with third-degree atrioventricular block by pacing His bundle and left bundle branch: Case report

**DOI:** 10.1097/MD.0000000000021097

**Published:** 2020-08-14

**Authors:** Denghong Zhang, Xiaoming Huang

**Affiliations:** a Department of Cardiology, Fifth People's Hospital of Chengdu; b Department of Surgical Intervention, People's Hospital of Wenjiang District, Chengdu City, China.

**Keywords:** atrial fibrillation, His bundle pacing, left bundle branch pacing, third degree atrioventricular block

## Abstract

**Introduction:**

Substantial advances in cardiac pacing technology have been developed in the past decades. However, efforts to improve pacing technology to achieve physiological electrical activity, such as with cardiac resynchronization therapy, are underway. Permanent His bundle pacing, which directly stimulates the His-Purkinje network and electrically activates both ventricles, simulates physiological electric activity in the heart, and has been considered an ideal pacing strategy to treat arrhythmias. For patients with atrial fibrillation complicated by third-degree atrioventricular block (AVB), permanent His bundle pacing is a better option than conventional right ventricular apical or septal pacing, the latter of which may be associated with risks, such as heart failure. However, His bundle pacing exhibits some shortcomings, including elevated pacing threshold, dislocation, and abnormal sensing.

**Case presentation:**

A 69-year-old female patient who had atrial fibrillation (AF) complicated by third-degree AVB and who was treated with permanent His bundle pacing combined with left bundle branch pacing.

**Diagnosis:**

AF complicated by third-degree AVB.

**Interventions:**

We used the left bundle branch as a backup pacing site to overcome any shortcomings related to permanent His bundle pacing.

**Outcomes:**

The patient recovered well without any events.

**Conclusion:**

We selected His bundle pacing as the primary pacing, but also used left bundle branch pacing as a backup approach. If His bundle pacing results in an increased sensing threshold, pacing threshold changes, or dislocations, left bundle branch pacing can compensate for dysfunction of permanent deficiencies in His bundle pacing, preserving physiological pacing.

## 1 Introduction

Cardiac pacing is the mainstay treatment for cardiac arrhythmia, particularly for persistent arrhythmias, such as irreversible bradyarrhythmia. Traditional right ventricular (RV) apical or septal pacing, while still widely used in the clinic, is associated with negative effects, including increased risk of atrial fibrillation (AF), impaired ventricular function, and heart failure.^[1]^ Currently, physiological pacing, such as cardiac resynchronization therapy (CRT), has shown substantial clinical advantages and is considered the future of developing cardiac pacing technology. Permanent His bundle pacing offers similar physiological stimulation by directly simulating the His-Purkinje network, electrically activating both ventricles, and thus reducing/eliminating dyssynchrony. Clinically, permanent His bundle pacing has been shown to maintain long-term cardiac function.^[2]^ However, permanent His bundle pacing exhibits some disadvantages, including higher capture threshold, progressively increasing threshold during long-term follow-up,^[3]^ and lead failure,^[4]^ In this report, we treated an AF patient with a third-degree atrioventricular block (AVB) with permanent His bundle pacing combined with left bundle branch pacing as a backup lead. The patient recovered with no major events during the 2-month follow-up period.

## 2 Case report

**Figure 1:**
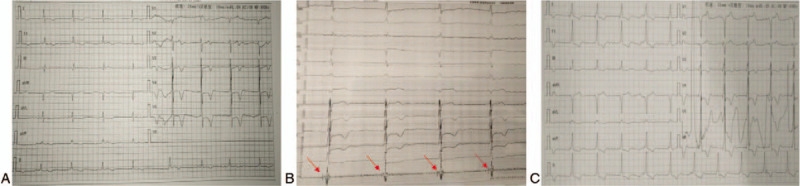
Representative electrocardiograms (ECGs) recorded before, during, and after operation. A, Preoperative baseline ECG: atrial fibrillation with intermittent third-degree AVB and ventricular rate of 48 bpm. B, Intraoperatively detected His bundle potential (indicated by the arrow) showing A to V separation and fixed H to V relationship, suggesting that the AVB was above the His bundle. C, ECG patterns before and after His bundle pacing are consistent in morphology. AVB = atrioventricular block.

A 69-year-old female patient was admitted to the hospital with fatigue and shortness of breath for 1 month. The patient had hypertension for 2 years but did not present with symptoms of syncope, chest tightness, or chest pain. Physical examination revealed the following: enlarged heart on the left side, heart rate of 46 beats per min (bpm), low heart sounds with no obvious pathological murmur, and level III NYHA cardiac function. After admission, dynamic electrocardiogram (ECG) revealed: AF with the fastest heart rate of 93 bpm and the slowest heart rate of 36 bpm (average: 47 bpm), ventricular escape beats, and intermittent third degree AVB (Fig. 1A). Echocardiography also showed a left atrial diameter of 38 mm, a left ventricular diastolic diameter of 48 mm, and a left ventricular ejection fraction of 80%. Full blood panel tests did not find any abnormality. The patient was diagnosed at admission with hypertension and AF combined with intermittent third-degree AVB. CRT implantation was not indicated since the ECG did not show a widened QRS and there was no decrease in left ventricular ejection fraction. We therefore implanted a double-chamber pacemaker (model: E50, Medtronic USA) on the His bundle. The His bundle potential showed separate A to V and fixed H to V relationships (Fig. 1B), suggesting that the AV block occurred within the AV node. We then performed His bundle pacing at the site that exhibited the clearest His bundle potential and implanted the Medtronic3830 pacing lead. The multi-indicator showed that the pacing lead recorded a clear His bundle potential with a pacing threshold of 1.0 V, pulse width of 0.4 ms, sensed amplitude of 5.5 V, impedance of 750 Ω, and QRS wave width of 98 ms after pacing, which was consistent with the preoperative QRS wave pattern (Fig. 1C). During the operation, the pacing threshold, impedance, and sensed parameters were repeatedly tested and deemed satisfactory.

**Figure 2:**
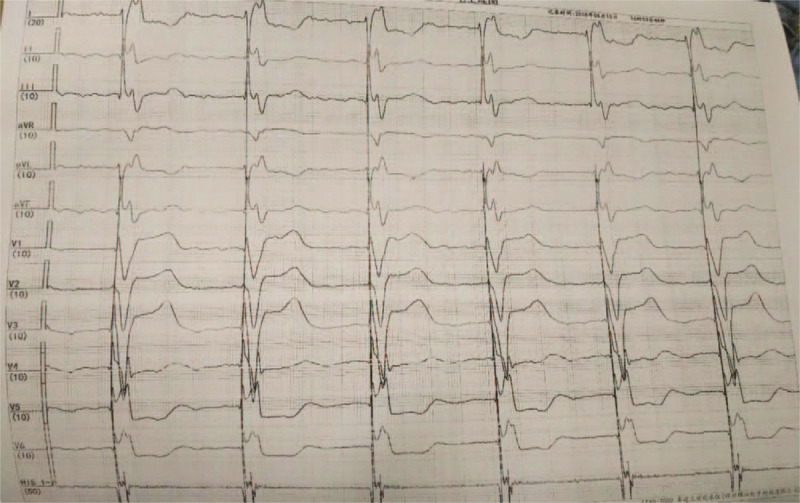
RV septal pacing ECG showing a complete left bundle branch block pattern. The V1 lead is QS-shaped; the V5, V6, I, AVL lead is widened to 160 ms with a notch on the top. ECG = electrocardiogram, RV = right ventricular.

**Figure 3:**
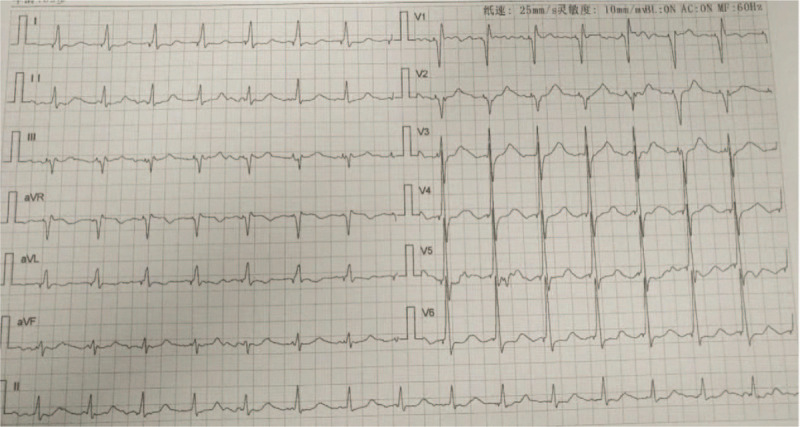
ECG after initiation of the left bundle branch pacing showing incomplete right bundle branch block. V1 lead shows a QR pattern; V5 and V6 leads show deepened S wave; the QRS width is 92 ms. ECG = electrocardiogram.

**Figure 4:**
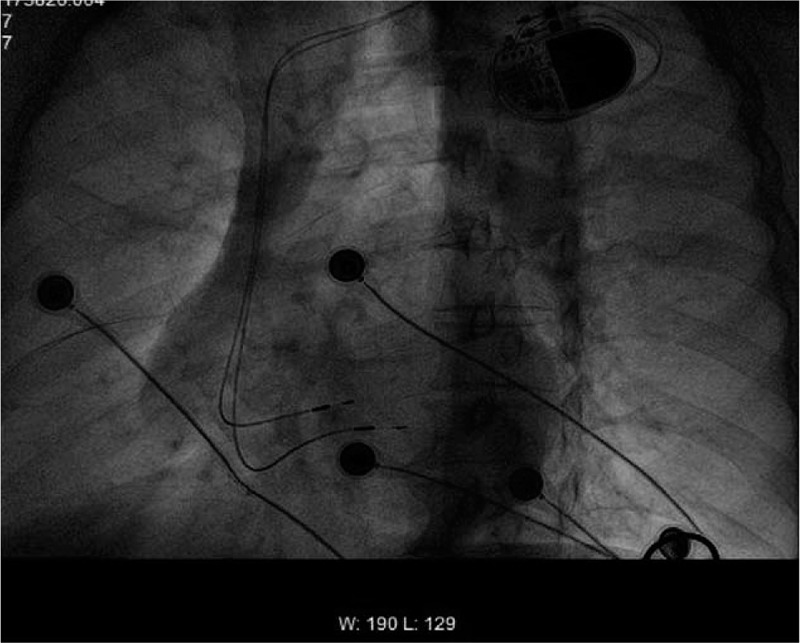
Lower left front radioscopy showing that the electrode lead points to the septum.

Next, we prepared the left bundle branch pacing for this patient as a backup lead. Under the 30° right anterior oblique projection position, another 3830 electrode was pushed to reach the RV septum, and was implanted into the position approximately 1 cm away from the His bundle lead. The electrode lead was positioned vertically toward the spine, indicating that the electrode lead was located at the RV septum. After pacing, a complete left bundle branch block was observed: the V1 lead was QS type, and the QRS waves on I and AVL leads were > 120 ms, with a notch on the top (Fig. 2). The 3830 electrode was then gradually screwed into the deep part of the interventricular septum. At this time, the multiple lead QRS waves suddenly narrowed to 92 ms, and the V1 lead developed into an incomplete right bundle branch block pattern: QR image, deepening S waves on V5 and V6 leads, and QRS width of 92 ms (Fig. 3), suggesting that the electrode lead entered the left bundle branch region of the left His-Purkinje system and was pacing. The electrode position was further examined via multiple radioscopic positions (Fig. 4), and the following parameters were obtained: pacing threshold of 1.2 V, sensed threshold of 15 V, impedance of 769 Ω, and no anterior diaphragm pacing detected at 10 V. Then, the His bundle electrode was connected to the atrial socket of the dual chamber pacemaker, the left bundle branch electrode was connected to the ventricular socket, and the pacemaker parameters were programmed with the following parameters: SAV of 200 ms, PAV of 220 ms, VSP off, Mode switch off, and a pulse width of 0.4 ms. The pacemaker was then placed into the position and the pocket was sealed with stitches. During 2 months of follow-up, the patient's clinical condition improved and the NYHA cardiac function level was restored to level II. The pacing threshold, pacing impedance, and sensing parameters of the His bundle and the left bundle branch were good (evidenced by a former pacing threshold of 0.9 V, sensed amplitude of 5.8 V, and impedance of 780 Ω, and a latter pacing threshold of 0.8 V, sensed amplitude of 18 V, and impedance of 770 Ω), and the patient did not report any cardiac events or symptoms.

## 3 Discussion

Traditional RV apical pacing is advantageous because it is a simple operation, involves easy placement, has a low dislocation rate, and has been successfully used to treat some arrhythmias, such as bradyarrhythmias and AV block, in heart failure patients.^[5]^ However, long-term follow-up data suggest that RV apical pacing changes the ventricular activation sequence, resulting in electrical and mechanical dyssynchronization between and within the ventricles, which may impair LV function and worsen heart failure.^[6]^ Thus, alternative pacing methods involving the RV septum, LV, outflow track, and biventricular pacing have been suggested.^[7,8]^

His bundle pacing has characteristics similar to that of physiological pacing, can reverse ventricular remodeling, restore ventricular electrical and mechanical synchrony, and subsequently improve heart function.^[9]^ However, a permanent His bundle pacing technique has several disadvantages, including poor sensing, a high pacing threshold, and a high long-term dislocation rate.^[10,11]^ Previously, left bundle branch pacing was used to successfully treat a patient with heart failure and left bundle branch block with the advantages of good sensing, a low pacing threshold, and a low risk of dislocation.^[12]^ In addition, left bundle branch pacing has a narrow QRS waveform and has better dual-chamber synchronization compared with conventional RV septal or apical pacing.^[12]^ In this case report, the patient had both AF and third-degree AVB. The ECG indicated that, after pacing the HIS bundle and the left bundle branch area, there was a very narrow QRS wave, indirectly indicating that the left and right ventricular synchronization was good. We selected His bundle pacing as the primary pacing, but also used left bundle branch pacing as a backup approach. If His bundle pacing results in an increased sensing threshold, pacing threshold changes, or dislocations, left bundle branch pacing can compensate for dysfunction of permanent deficiencies in His bundle pacing, preserving physiological pacing. Following the implant, the patient recovered well with no major events during the 2-month follow-up period. Going forward, we will alternate the use of 2 pacing methods, continue to observe ECG and NYHA cardiac function changes, and compare the advantages and disadvantages of the 2 pacing methods. However, a longer follow-up period is needed to further determine if the pacing techniques we employed are safe and reliable. This case study offers evidence for developing new technical approaches for His bundle pacing treatment of AF patients in the clinic.

## Author contributions

XXXX.
